# Supplementary material for: Assessing Genomic Mutations in SARS-CoV-2: Potential Resistance to Antiviral Drugs in Viral Populations from Untreated COVID-19 Patients
Source: Microorganisms. 2023 Dec 19;12(1):2. doi: 10.3390/microorganisms12010002 (PMC10821222; doi:10.3390/microorganisms12010002)
Supplement: Supplementary file 1 [file microorganisms-12-00002-s001.zip › Table_S1_GISAID.pdf]

SUPPLEMENTAL TABLE1

Data Availability

GISAID Identifier: EPI_SET_231207zf
doi: 10.55876/gis8.231207zf

All genome sequences and associated metadata in this dataset are published in GISAID's EpiCoV database. To view the contributors of each individual sequence with details such as accession number, Virus name, Collection date, Originating Lab and Submitting Lab and the list of Authors, visit 10.55876/gis8.231207zf

Data Snapshot

- EPI_SET_231207zf is composed of 4,156 individual genome sequences.
- The collection dates range from 2021-04-05 to 2022-10-05;
- Data were collected in 1 countries and territories;
- All sequences in this dataset are compared relative to hCoV-19/Wuhan/WIV04/2019 (WIV04), the official reference sequence employed by GISAID (EPI_ISL_402124). Learn more at https://gisaid.org/WIV04.
